# Evolution of prostate MRI: from multiparametric standard to *less-is-better* and *different-is better* strategies

**DOI:** 10.1186/s41747-019-0088-3

**Published:** 2019-01-28

**Authors:** Rossano Girometti, Lorenzo Cereser, Filippo Bonato, Chiara Zuiani

**Affiliations:** Institute of Radiology, Department of Medicine, University of Udine – University Hospital “S. Maria della Misericordia”, p.le S. Maria della Misericordia, 15–33100 Udine, Italy

**Keywords:** Contrast media, Magnetic resonance imaging, Positron-emission tomography, Prostate imaging reporting and data system (PI-RADS), Prostatic neoplasms

## Abstract

Multiparametric magnetic resonance imaging (mpMRI) has become the standard of care to achieve accurate and reproducible diagnosis of prostate cancer. However, mpMRI is quite demanding in terms of technical rigour, patient’s tolerability and safety, expertise in interpretation, and costs. This paper reviews the main technical strategies proposed as *less-is-better* solutions for clinical practice (non-contrast biparametric MRI, reduction of acquisition time, abbreviated protocols, computer-aided diagnosis systems), discussing them in the light of the available evidence and of the concurrent evolution of Prostate Imaging Reporting and Data System (PI-RADS). We also summarised research results on those advanced techniques representing an alternative *different-is-better* line of the still ongoing evolution of prostate MRI (quantitative diffusion-weighted imaging, quantitative dynamic contrast enhancement, intravoxel incoherent motion, diffusion tensor imaging, diffusional kurtosis imaging, restriction spectrum imaging, radiomics analysis, hybrid positron emission tomography/MRI).

## Key points

- Multiparametric magnetic resonance imaging is the standard of care for assessing prostate cancer.
- Alternative protocols are emerging to increase availability and offer a patient-centred approach.
- *Less-is-better* strategies are promising for clinical practice, but require validation.
- *Different-is-better* strategies are a matter for intensive research.
- Prostate MRI technical standard and interpretation rules are still evolving.

## Background

Until recent times, prostate magnetic resonance imaging (MRI) was a poorly available examination reserved to stage prostate cancer (PCa). Enormous advances in MRI technology and wider availability of 3-T magnets contributed to an ever-increasing demand for the examination, as supported by the evidence-based expansion of indications. Prostate MRI is becoming of central importance in the contemporary management of PCa by improving the detection of clinically significant cancer (csPCa) while minimising overdiagnosis and overtreatment of indolent disease [1]. MRI is gaining acceptance in detecting and localising csPCa lesions, triaging biopsy, guiding targeted biopsy or focal therapy, stratifying the risk before treatment, monitoring patients during active surveillance, planning and choosing surgery or radiation therapy techniques, and assessing recurrence [2–8].

Expansion of prostate MRI has been accompanied by the definition of a substantially standardised examination technique named multiparametric MRI (mpMRI) [9, 10], providing anatomic, functional, and physiologic parameters for image analysis. However, mpMRI is a demanding approach in terms of execution, patient’s tolerability and safety, expertise in interpretation, and costs, as we will discuss in this review. Clinical practice and research are soliciting the adoption of different MRI protocols to face the above challenges, following a general *less-is-better* strategy leading to a faster and cheaper examination in which essential parameters are retained for analysis.

In this review, we describe the current multiparametric standard for prostate MRI, together with the limitations inherent to its ongoing evolution. We also discuss *less-is-better* strategies as potential solutions according to the available literature and also describe novel advanced techniques for image acquisition and/or interpretation that can be considered as *different-is-better* strategies.

## The multiparametric standard

### mpMRI: towards simplification

The first attempt to establish minimal technical requirements for mpMRI came in 2012 from the European Society of Urogenital Radiology (ESUR) guidelines, which defined mpMRI as the combination of anatomic T2-weighted imaging (T2WI) with at least two functional MRI techniques [9]. The definition was supported by previous studies showing that two functional techniques complement T2WI better than one in terms of lesions characterisation, with diffusion-weighted imaging (DWI) and magnetic resonance spectroscopic imaging (MRSI) improving specificity and dynamic contrast-enhanced (DCE) imaging improving sensitivity [9]. ESUR guidelines proposed detailed and stringent technical requirements for detection and staging, with DWI and DCE to be used mandatorily and MRSI optionally. Since the guidelines were intended as mean to standardise imaging acquisition, interpretation, and reporting, protocols were presented together with the first version of the prostate imaging reporting and data system (PI-RADS), in which each of the sequences was scored separately to assess the risk that an MRI finding was a csPCa.

**Fig. 1:**
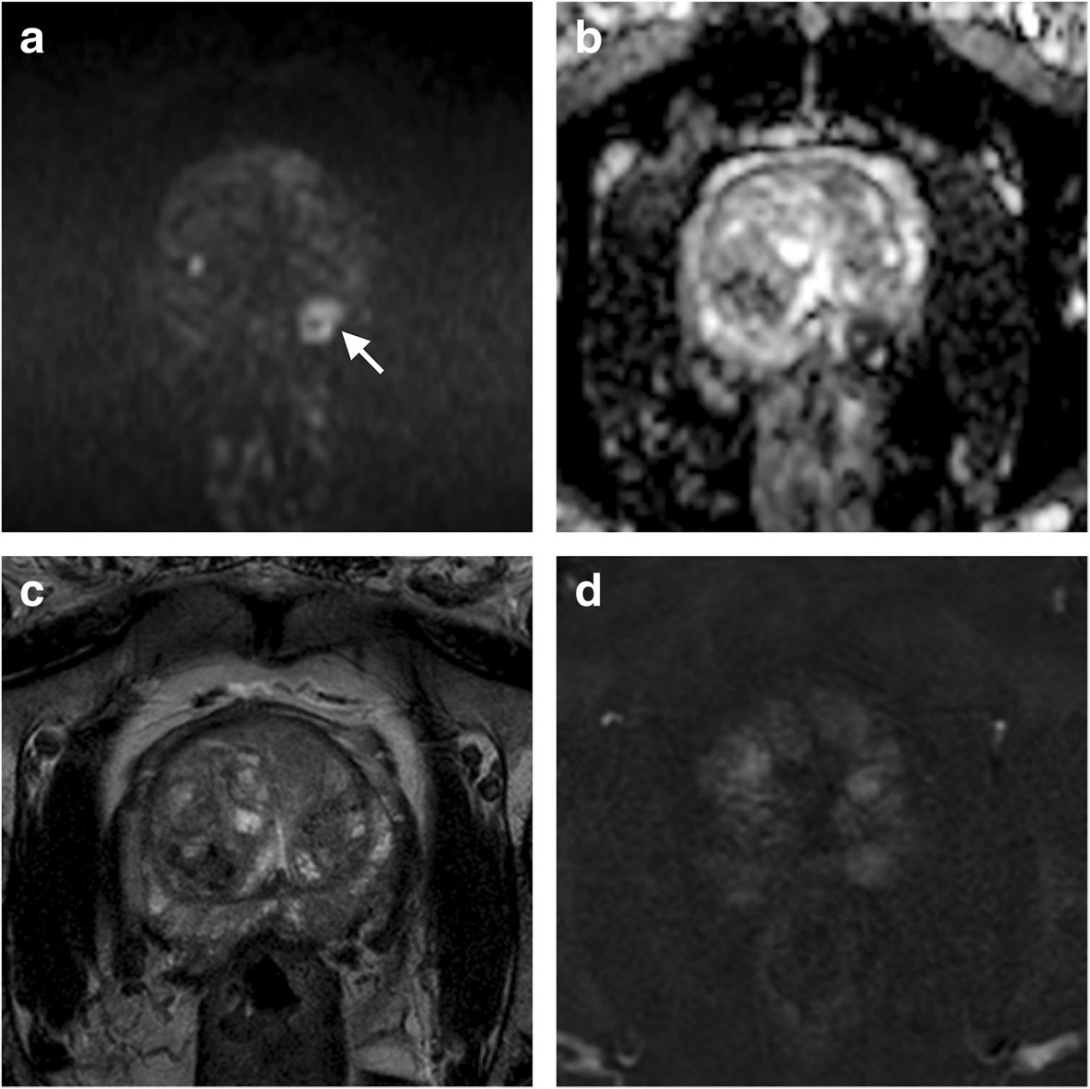
Application of the Prostate Imaging Reporting and Data System (PI-RADS) version 2 in interpreting a finding in the peripheral zone (PZ) of a biopsy-naïve 62-year-old man undergoing prostate multiparametric magnetic resonance imaging (mpMRI) for elevated prostate specific antigen (PSA) level. The dominant sequence, *i.e.*, a transverse diffusion-weighted imaging (DWI) sequence, showed an area (< 15 mm in size) of restricted water diffusion in the left midgland PZ, as testified by high signal intensity on the *b* = 2000 s/mm^2^ image (arrow in **a**) and corresponding marked hypointensity on the apparent diffusion coefficient (ADC) map (**b**). This finding was classified as PI-RADS 4 accordingly, with additional ancillary suspicious features such as hypointensity on transverse T2-weighted imaging (**c**) and focal early contrast enhancement on subtracted T1-weighted imaging (**d**). Pathology after radical prostatectomy found a Gleason score 7 (3 + 4) T3a N0 cancer

**Fig. 2:**
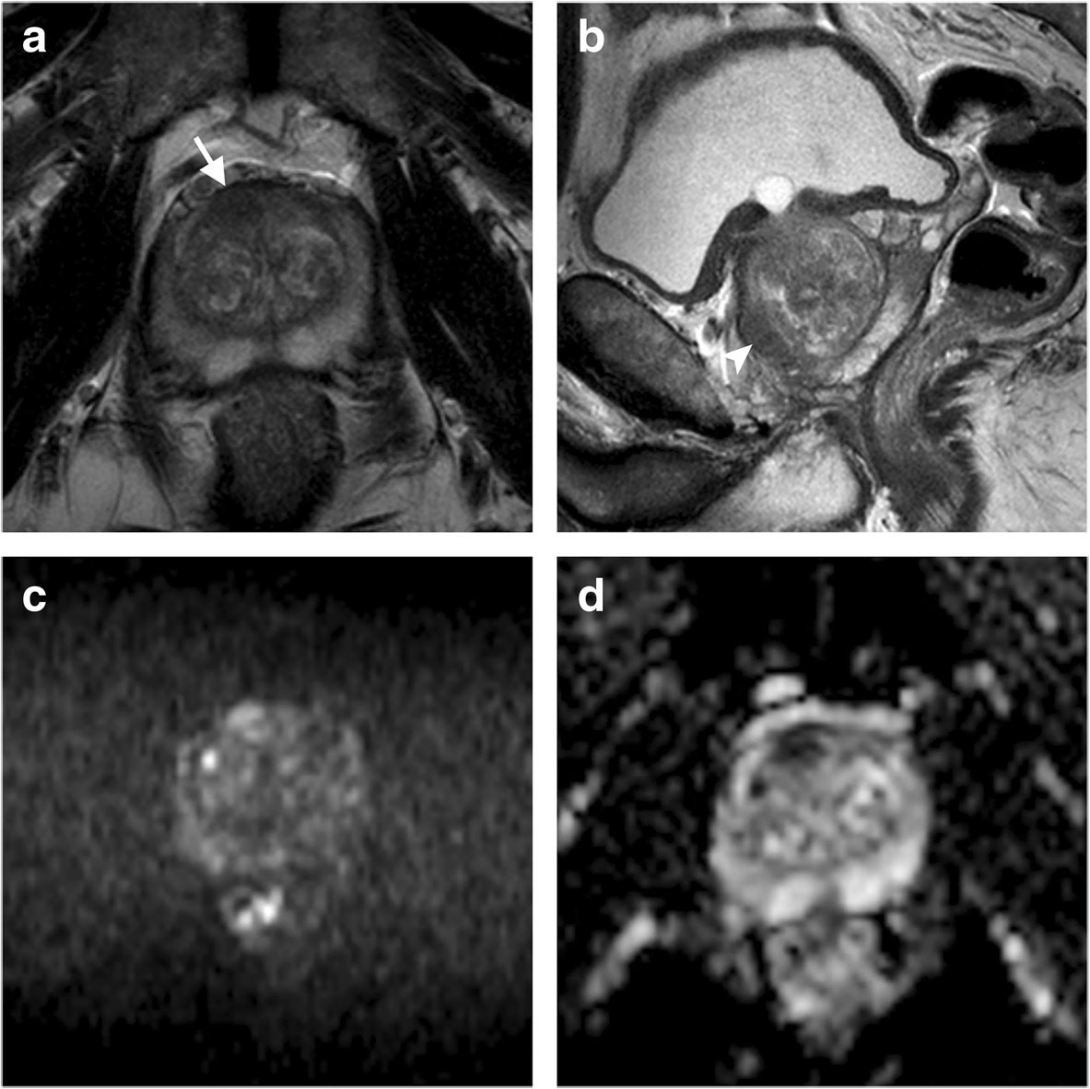
Use of PI-RADS version 2 criteria to categorise a transition zone (TZ) finding in a 67-year-old patient with elevated PSA (7.80 ng/mL) and previous negative biopsies. On transverse (arrow in **a**) and sagittal (arrowhead in **b**) T2-weighted images, there was an anterior, right-sided TZ focal area with lenticular shape, ill-defined margins, and capsular bulging (< 15 mm in size), close to the anterior fibromuscular stroma. This finding was scored as PI-RADS 4 and confirmed to be a Gleason score 6 (3 + 3) cancer on biopsy and subsequent radical prostatectomy (T2b N0). DWI supported the suspicion of malignancy by showing restricted diffusion as corresponding, focal hyperintensity on *b* = 2000 s/mm^2^ image (**c**) and marked hypointensity on the ADC map (**d**)

The second version of the guidelines (PI-RADS version 2) was updated in late 2014 by a Steering Committee established the American College of Radiology, ESUR, and the AdMeTech Foundation [10]. PI-RADS version 2 led to consistent simplification of several technical and interpretation aspects compared to PI-RADS version 1. First, MRSI was excluded from the examination, restricting the multiparametric standard to the use of T2WI, DWI, and DCE. MRSI was classified as an advanced research tool, thus recognising the impractical use in everyday clinical practice. Accordingly, the PI-RADS version 2 score no longer included qualitative and quantitative assessment of tissue choline and citrate. Second, PI-RADS version 2 proposed less (even if stringent) technical parameters to obtain an acceptable mpMRI examination, leaving space for protocols optimisation based on the available equipment, as well as for tailoring MRI protocols on the basis of clinical questions and patients’ characteristics. Finally, the interpretation system was modified by introducing the concept of *dominant sequence*, according to which the likelihood that an image finding represents csPCa, expressed on a 1 to 5 scale, mainly depends on its appearance on DWI for the peripheral zone (PZ) (Fig. 1) and on T2WI for the transition zone (TZ) (Fig. 2) (Table 1). DCE was assigned a secondary role, *i.e.*, to act as a tiebreaker to upgrade to category PI-RADS 4 those PZ findings initially categorised as PI-RADS 3, when they exhibit focal early contrast enhancement on visual analysis [10]. Similarly, DWI was identified as the tiebreaker for TZ findings with ambiguous appearance on T2WI. This approach no longer included the quantitative evaluation of the apparent diffusion coefficient (ADC) as a contributor to PI-RADS categorisation.

**Table 1:** PI-RADS version 2 interpretation rules for mpMRI to detect csPCa

Peripheral zone		PI-RADS category	Transition zone	
Finding appearance on DWI	DCE		DWI	Finding appearance on T2WI
Score 1: no abnormalities on ADC and high *b* value DWI	–	1	–	Score 1: Uniform hyperintense signal intensity
Score 2: indistinct hypointensity on ADC	–	2	–	Score 2: Linear or wedge-shaped hypointensity or diffuse mild hypointensity
Score 3: focal hypointensity on ADC and isointensity on high *b* value DWI	No focal and early enhancement	3	If DWI score ≤ 4	Score 3: Heterogeneous signal intensity or non-circumscribed, rounded, moderate hypointensity
	Focal and early enhancement	4	If DWI score = 5	
Score 4: focal markedly hypointensity on ADC and markedly hyperintensity on high *b* value DWI	–	4	–	Score 4: Circumscribed, homogenous moderate hypointense focus/mass confined to prostate, and < 1.5 cm in greatest dimension
Score 5: same as 4 but ≥ 1.5 cm in greatest dimension or definite extraprostatic extension/invasive behaviour	–	5	–	Score 5: Same as 4 but ≥ 1.5 cm in greatest dimension or definite extraprostatic extension/invasive behaviour

*csPCa* Clinical significant prostate cancer, *DCE* dynamic contrast-enhanced imaging, *DWI* diffusion-weighted imaging, *PI-RADS* Prostate Imaging Reporting and Data System, *T2WI* T2-weighted imaging. Probability of csPCa: category 1 = very low, category 2 = low, category 3 = intermediate/equivocal, category 4 = high, category 4 = very high

Moreover, the semiquantitative assessment of the enhancement curves (type 1, type 2, and type 3) proposed by the PI-RADS version1 was abandoned in favour of a simpler dichotomic rule (presence versus absence of focal enhancement of the category 3 finding), as supported by the evidence that visual analysis performs similarly to semiquantitative methods [11]. PI-RADS version 2 will be used in this review as the ideal framework to discuss the technical standard of mpMRI, though the document does not cover the whole spectrum of indications (*e.g.*, PI-RADS categorisation does not apply to the evaluation of recurrent disease or progression during surveillance). State-of-the-art mpMRI should include T2WI, DWI, and DCE T1-weighted imaging, whose mail technical aspects are summarised in Table 2 [10, 12–14]. DCE-unrelated T1-weighted imaging is also part of mpMRI, showing an ancillary role in assessing regional anatomy, post-biopsy changes, as well as the nodal status and possible presence of metastatic bone lesions. Of note, 3-T field strength and/or endorectal coils are no longer considered indispensable for mpMRI if protocols are adequately optimised [10, 12].

**Table 2:** Main technical aspects and rationale for mpMRI core-sequences

Sequence	Rationale/investigated parameter	Technique	Role in prostate cancer assessment with mpMRI	Limitations
DWI	To exploit restricted diffusion of water molecules as a marker of increased cellularity and neoplastic reorganisation of normal glandular tissue	• Fat-saturated, free-breathing single-shot spin-echo echo-planar imaging • At least two *b* values to generate the ADC map (*e.g.*, minimum 50–100 s/mm^2^, maximum 800–1000 s/mm^2^); extrapolated ultra-high b-values (≥ 1400 s/mm^2^) can also be used to generate the ADC map • Ultra-high *b* values can be acquired to increase tumour conspicuity (not for the ADC map generation in less performing systems) • Field of view 16–22 cm, slice thickness ≤ 4 mm without gap, pixel size ≤ 2.5 mm (phase and frequency), TR ≤ 3000 ms, TE ≤ 90 ms	Detection and localisation: • dominant sequence for assessing PZ findings • secondary role in assessing category 3 findings found by T2WI in the TZ	• Sensitive to artefacts from air in the rectum and/or motion • Distortions • Relatively unstandardised technique, leading to limited reproducibility of the quantitative analysis of ADC (no definite cut-off values) • Significant overlap of ADC values between benign conditions and tumours with different aggressiveness
T2WI	To provide high-resolution and high-contrast representation of the zonal anatomy of the prostate, as well as of periprostatic anatomy (seminal vesicles, neurovascular bundles, bladder, rectum, and the levator ani)	• 2D turbo spin-echo with high spatial resolution: field of view 12–20 cm to cover the prostate and the seminal vesicles; slice thickness ≤ 3 mm with no gap; pixel size ≤ 0.7 mm (phase) *x* ≤ 0.4 mm (frequency) • Sagittal, oblique transverse, oblique coronal (posterior prostate wall as anatomic landmark)	• Detection and localisation: dominant sequence for assessing TZ findings • Locoregional staging: detection of extraprostatic extension or seminal vesicle invasion	• Nonspecific tumour appearance, overlapping with that of non-malignant conditions (*e.g.*, inflammation or post-biopsy changes) • Sensitive to motion artefacts given the prolonged acquisition time
DCE	To detect earlier and more intense contrast enhancement of cancer compared to normal prostatic tissue, as the expression of tumoural neoangiogenesis (denser, poorly formed vessels with increased capillary permeability)	• Sequential acquisition of a T1-weighted 2D or 3D gradient-echo sequence with high temporal resolution (≤ 10 s, ideally ≤ 7 s, with TR < 5 ms and TE < 100 ms). Acquisition before, during and after contrast injection (at least 2 min) to detect early enhancement • Field of view encompassing the whole gland and seminal vesicles • Slice thickness ≤ 3 mm without gap, and pixel size ≤ 2 mm (phase and frequency) • If possible fat-saturated or subtracted images • Oblique transverse plane • Contrast injection rate 2–3 mL/s	• To upgrade ambiguous findings in the PZ • See Table 3 for further details	• Variable enhancement pattern of cancer, overlapping with non-malignant conditions (*e.g.*, inflammation or benign prostatic hyperplasia) • Longer acquisition time (> 2 min) to assess the permeability

*2D* two-dimensional, *3D* three-dimensional, *ADC* apparent diffusion coefficient, *DCE* dynamic contrast-enhanced imaging, *DWI* diffusion-weighted imaging, *mpMRI* multiparametric magnetic resonance imaging, *PZ* peripheral zone, *T1WI* T1-weighted imaging, *T2WI* T2-weighted imaging, *TE* time of echo, *TR* time of repetition, *TZ* transition zone

### Disadvantages of mpMRI

PI-RADS version 1 and version 2 were elaborated as expert-consensus documents needing subsequent clinical validation. PI-RADS version 2 guideline reported a high pooled sensitivity (0.85–0.89) and an acceptable pooled specificity (0.71–0.79) for PCa according to meta-analyses [15, 16]. The proposed methodology was found to be effective as a risk stratification tool, with cancer rates for PI-RADS categories 3, 4, and 5 of 33.1%, 70.5%, and 90.7%, respectively [17].

However, PI-RADS version 2 suffers from several weak points [1], including questioned interpretation criteria for TZ cancers [18], lack of definite rules for the central zone or anterior fibromuscular stroma involvement [1], and moderate inter-reader agreement [19], especially for TZ assignments and DCE [20]. In general, there is a relatively low positive predictive value for category 3 findings, a false-negative rate for csPCa ranging typically from 5 to 15%, and a false-positive rate up to 60–80% for PI-RADS 4 lesions in some series [3]. While PI-RADS version 2 shows limited sensitivity for less relevant cancers (*e.g.*, low-risk Gleason score 3 + 3 lesions with tumour volume lower than 0.5 mL), index csPCa lesions with a Gleason score equal to or greater than 3 + 4 and a volume equal to or greater than 0.2 mL (*i.e.*, about 7–8 mm in size) can be detected [1]. However, some series found limited accuracy for csPCa (*i.e.*, with Gleason score 4 + 3 or higher) with a tumour volume equal to or lower than 0.5 mL [21].

Not surprisingly, PI-RADS version 2 is a *living document*, with the incoming 2.1 version aiming to refine mpMRI categorisation (*e.g.*, size cut-off for categories 4 and 5, role for DWI and DCE in the TZ) and improved inter-reader agreement [1].

From a technical point of view, there are some disadvantages as mpMRI stands. First, the examination is poorly patient-centred, because it presents as a *plug-and-play* tool that does not account for different scenarios of application (*e.g.*, clinical research in tertiary referral or academic centres versus clinical routine) or specific clinical questions (*e.g.*, detection in men with elevated PSA level, staging, or active surveillance). Additionally, mpMRI requires prolonged time in the magnet (up to 30–45 min [22]), which has been identified as the major source of stress in patients undergoing MRI [23]. The use of endorectal coil can further emphasise this aspect [24], suggesting that the examination is far from being perfect in terms of patient’s tolerability.

Second, the use of intravenous gadolinium-based contrast agents is associated to the risk of adverse events such as allergic-like/hypersensitivity reactions and nephrogenic systemic fibrosis in patients with advanced chronic kidney disease, as well as to gadolinium deposition in the brain of subjects undergoing repeated exposure [25]. Although the frequency and clinical relevance of those conditions is a matter of debate [25], radiologists should take care in identifying patients at risk and avoid unjustified contrast-related risks in those cases where DCE is not supposed to provide any added value. One should also take into account those complications occurring after the insertion of a peripheral intravenous catheter such as discomfort and phlebitis, which—though mild in nature—show a frequency up to 27% in some series [26].

Finally, mpMRI implies high examination- and patient management-related costs, which are a relevant factor in determining whether to implement it systematically in a clinical setting [27]. Theoretically, mpMRI can save the costs related to inconclusive diagnoses (*e.g.*, in patients with cancer and repeated negative biopsies) or unnecessary biopsy [28] and was recently shown to be cost-effective as the first test for PCa diagnosis [5, 29]. However, it is still unclear how to refine or change the mpMRI standard in order to balance cost saving with the diagnostic accuracy required by different clinical scenarios such as initial diagnosis, fusion biopsy, active surveillance, staging, or recurrence.

## *Less-is-better* strategies

Different strategies are emerging as a potential solution for the limitations and challenges discussed above. They can be qualified as various forms of a *less-is-better* approach, in which one or more aspects of current mpMRI are considered redundant, especially in the PCa detection setting, thus being eliminated or changed as discussed below.

### Non-contrast biparametric MRI

Whether DCE should be included in the multiparametric standard has always been a controversial issue [28], showing both supporters and opposers. Pros and cons of DCE are summarised in Table 3 [13, 28, 30–33].

**Table 3:** Pros and cons of performing DCE imaging in prostate mpMRI compared to the combination of T2WI and DWI

	Pros	Cons
Detection and localisation	Gain in sensitivity for cancers located in hypovascular and fibrous zones (anterior fibromuscular stroma, central zone) or showing challenging appearance such as non-nodular infiltrating lesions in the peripheral zone	Gain in sensitivity compared to T2WI alone, but no added value compared to T2WI and DWI
	Gain in specificity (up to 17%) in differentiating cancer from atrophy, necrosis, haemorrhage, prostatitis, calcifications	Variable enhancement patterns in cancer, overlapping with benign conditions
	Problem solver in PI-RADS version 2 for peripheral zone lesions	–
	Rescue of examinations with inadequate or absent T2WI and/or DWI	–
	Primary role in detecting recurrence after treatment	–
	Research: prediction of tumour volume, prediction of biological aggressiveness (microvessel tissue density or Gleason score)	–
Staging	Gain in accuracy in less experienced readers (“first localise, then stage” approach), especially for seminal vesicle invasion	Conflicting results in literature
	Gain in assessing extraprostatic extension by detecting extraprostatic contrast enhancement	False positives related to inflammation
Patient-centred care	Negligible extra time in magnet	Extra time in magnet reducing patient comfort and compliance
	Adverse reactions to gadolinium-based contrast agents are rare and usually of limited clinical significance	Safety issues related to gadolinium-based contrast agents, including adverse reactions and gadolinium deposition in the brain
Costs	–	Increased costs (up to 20–30% of the whole examination).

See references: [13, 28, 30–33]. *DCE* dynamic contrast-enhanced, *DWI* diffusion-weighted imaging, *mpMRI* multiparametric magnetic resonance imaging, *PI-RADS* Prostate Imaging Reporting and Data System, *T2WI* T2-weighted imaging

DCE has been classically assumed to improve the sensitivity of T2WI alone or T2WI combined with DWI, based on studies showing an average added value of about 10–15% [28]. While acknowledging this, opposers argue that this technique is redundant in most examinations, since it improves T2WI alone, while showing no relevant added value compared to the combination of T2WI and DWI [13]. This statement is supported by the results of several single-centre studies [34–36] and of a recent meta-analysis [37], as well as by the empirical experience from large-volume centres. Furthermore, a csPCa can present with different contrast enhancement patterns, overlapping at a significant extent with those of other benign conditions such as prostatitis or benign hypertrophy nodules in the TZ [13]. As aforementioned, PI-RADS version 2 acknowledged those limitations by circumscribing the role for DCE to PZ only and limiting it to a qualitative evaluation on a binary base for problem-solving purpose [10].

**Fig. 3:**
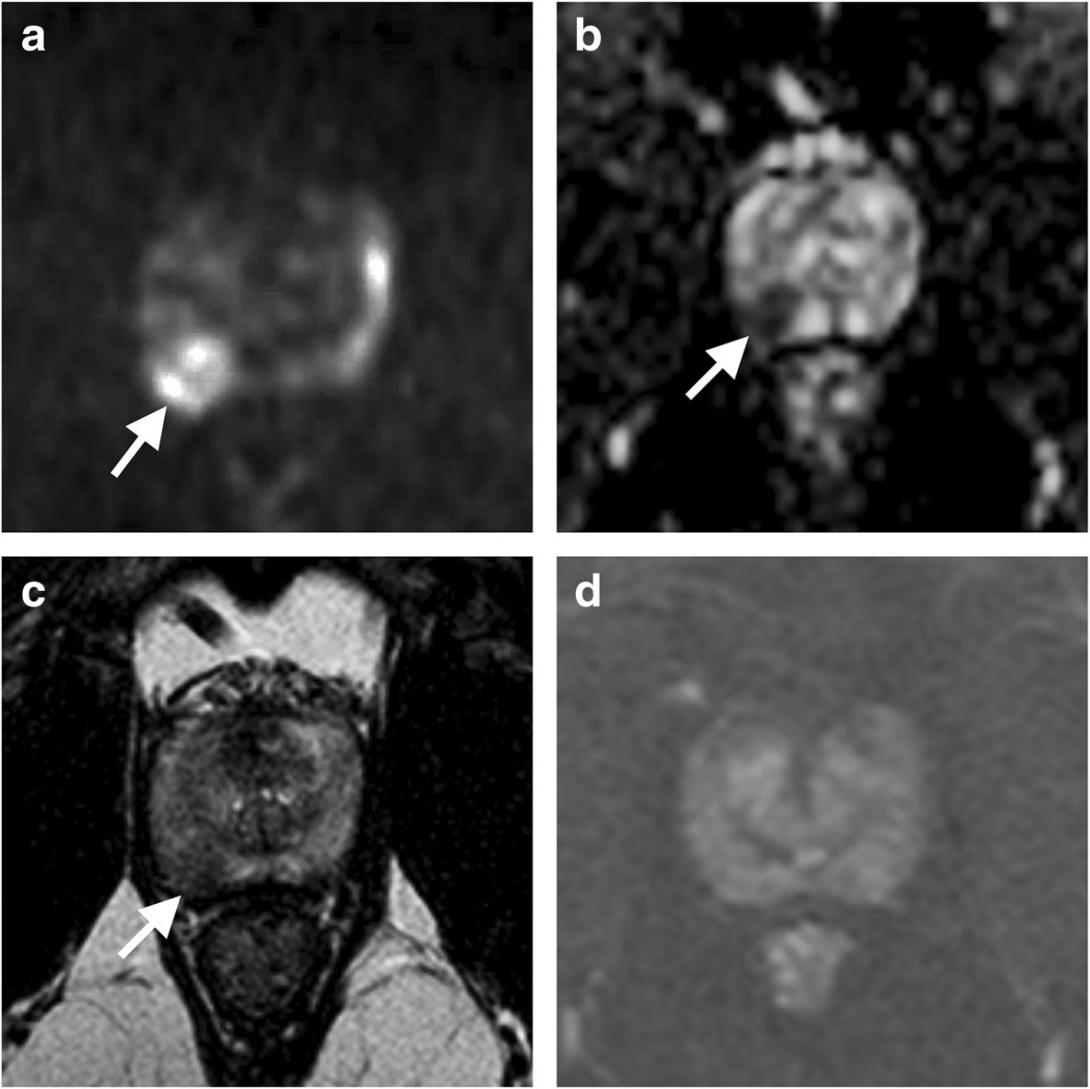
Example case of mpMRI in which the information given by dynamic contrast-enhanced (DCE) imaging was redundant. A 54-year-old man with slightly elevated PSA level (3.43 ng/mL) and suspicious digital rectal examination underwent the examination to target biopsy, showing right midglandular < 15 mm in size PZ finding categorised as PI-RADS 4 because of focal restricted diffusion well visible as hyperintensity on the high *b* value image (**a**) and hypointensity on the ADC map (**b**), associated to hypointensity on a transverse T2-weighted image (arrows in **c**). Cancer was proven by biopsy and then by pathology after surgery with Gleason score 7 (4 + 3). Of note, transverse subtracted DCE (**d**) had no role in detecting and localising cancer, showing no differences in contrast enhancement compared to the surrounding PZ

The MRI protocol excluding DCE is usually named *biparametric MRI* (bpMRI), being composed of anatomic T2WI coupled with DWI as the only retained functional technique (Fig. 3). This simplified approach is ever-increasingly used in clinical practice for the detection/localisation or staging of csPCa lesions, as testified by its incorporation into some guidelines as the technical standard for those indications [38]. In this approach, DCE is only used in the case of non-diagnostic DWI and/or T2WI, as happens because of artefacts from motion, air in the rectum, or hip prosthesis.

How does bpMRI work in detecting and localising cancer? A myriad of studies tried to answer this question, using PI-RADS or different interpretation criteria [39]. A meta-analysis by Woo et al. [40] focused on head-to-head comparisons between bpMRI and mpMRI, including 2142 patients from 20 papers. The analysis found comparable pooled sensitivity (0.74 *versus* 0.76), specificity (0.90 *versus* 0.89), and area under the curve (0.90 *versus* 0.90), without clinically relevant or statistically significant differences at subgroup analysis stratifying for a variety of factors, including field strength (1.5 T versus 3.0 T), type of standard of reference (radical prostatectomy *versus* biopsy), PI-RADS version (version 1 *versus* version 2), or type of DCE analysis (qualitative, semiquantitative, or quantitative). Of note, most of the included studies were based on small retrospective cohorts, showing great heterogeneity and a variable definition of csPCa. Thus, bpMRI should be validated with large, prospective multicentric trials aimed to confirm whether the two technical approaches are really equally effective in different clinical scenarios, and the rate of cancers missed by avoiding DCE is as minimal as reported in previous studies, *e.g.* by Vargas et al. [21] (4 out of 125).

A recent paper by Junker et al. [30] raised the question of which interpretation rules should be used with bpMRI. Those authors compared 236 bpMRI and mpMRI readings performed by a single experienced observer within a unique reading session, showing that 94.1% of cancers were scored identically using PI-RADS version 2 rules. Assuming that PI-RADS 3 findings in the PZ could not be further upgraded with bpMRI, the above authors observed a shift from category 4 to category 3 in 5.9% of cases when moving from mpMRI to bpMRI. All those findings included low-risk cancers with a predominant Gleason score 3 pattern, suggesting that a few cancers with limited clinical significance were missed by bpMRI. However, the majority of category 3 findings do not harbour malignancy [17], thus posing the problem of how to manage them [30, 41] and emphasising the risk that bpMRI can induce an increased number of unnecessary biopsies. To overcome this problem, different rules for upgrading category 3 findings have been proposed, including low ADC values [41] or volumes equal to or greater than 0.5 cm^3^ [42]. However, those methods are prone to low reproducibility. Rules for upgrading and managing category 3 lesions are still a matter of debate and will be probably refined by future PI-RADS versions.

Importantly, bpMRI does not apply to the setting of tumour recurrence after radical prostatectomy, radiation therapy, or focal therapy. DCE still plays a key-role in this scenario, as contrast enhancement is one of the most reliable features of disease in a context in which the prostate is no longer present or shows relevant therapy-induced changes making the PI-RADS inapplicable. The imaging of PCa recurrence is beyond the purpose of this review and has been treated comprehensively elsewhere [8].

### Reduced acquisition time

**Fig. 4:**
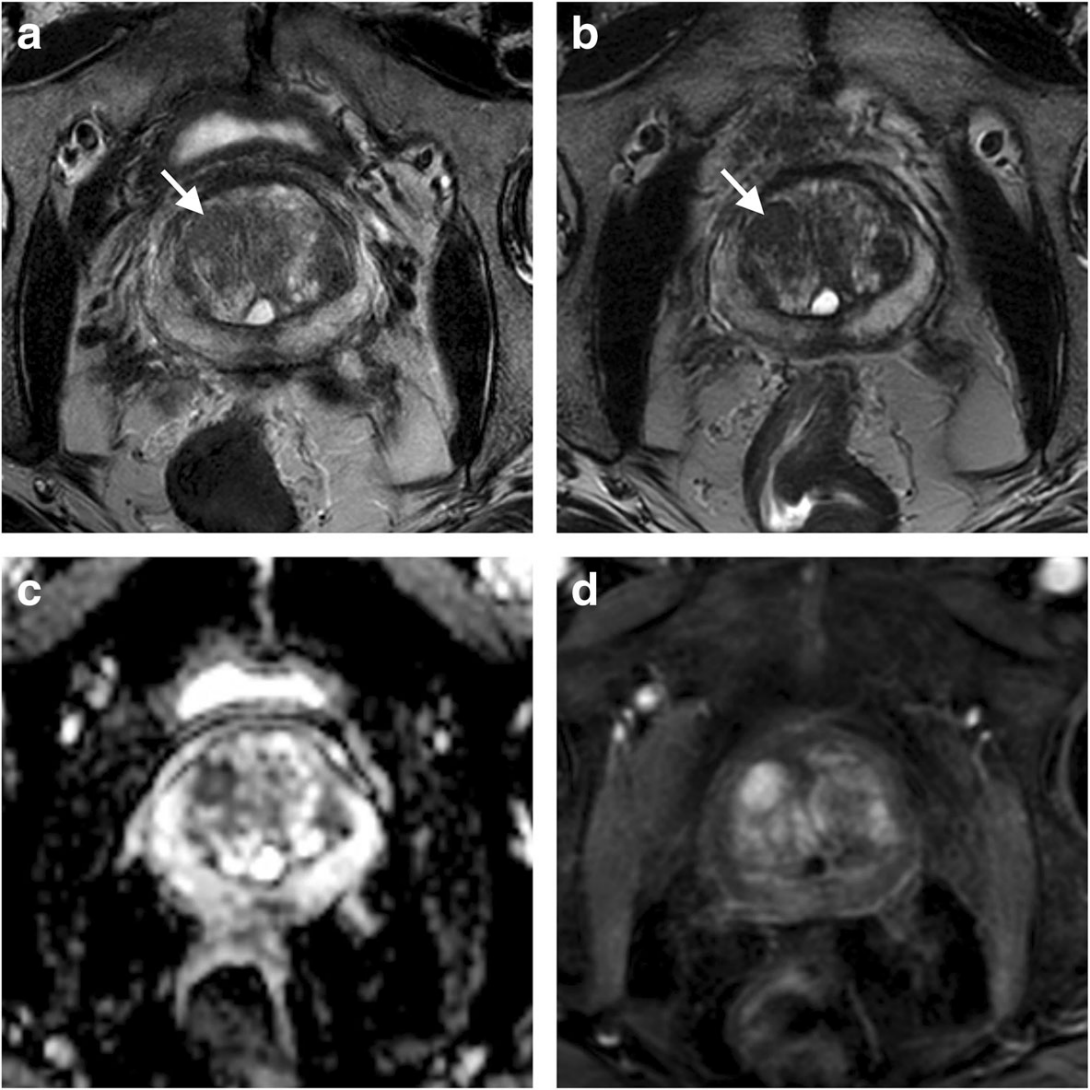
Three-dimensional (3D) T2-weighted imaging in a biopsy-naïve 69-year-old patient with elevated PSA (5.39 ng/mL) undergoing mpMRI for the purpose of targeted biopsy. The examination showed a nodule in the right TZ showing moderate hypointensity on transverse two-dimensional T2-weighted imaging and somewhat ill-defined margins (arrow in **a**). 3D T2-weighted imaging better delineated the nodule margins as a “charcoal” peripheral rim (arrow in **b**) by reducing image blurring, thus contributing to characterise it as a benign prostate hyperthropy (BPH) fibrostromal nodule. The nodule showed restricted diffusion (hypointensity on the ADC map in **c**) and intense, early contrast enhancement on transverse fat-saturated T1-weighted imaging (**d**)

T2WI is obtained with two dimensional (2D) turbo (or fast) spin-echo sequences, which usually require long acquisition time. This makes T2WI the main time-consuming phase of the examination, given the need to acquire transverse, sagittal, and coronal planes separately. Alternatively, three-dimensional (3D) volumetric T2WI provides a unique slab with isotropic voxels (*e.g.*, 0.8 × 0.8 × 0.8 mm) to be reconstructed in any plane, thus shortening the acquisition time up to 44% [43, 44]. Additionally, 3D T2WI is supposed to reduce volume-averaging artefacts, leading to better delineation of subtle anatomic features affecting the diagnosis at a relevant extent (*e.g.*, the so-called “erased charcoal sign” around TZ nodules, or prostate capsule integrity) (Fig. 4) [12].

Using a special sequence named “sampling perfection with application-optimised contrast using different flip-angle evolutions” (SPACE), Polanec et al. [44] recently showed similar accuracy between 2D T2WI and 3D T2WI in assessing PCa with PI-RADS version 2 criteria. This sequence was found to provide similar results to those given by 2DT2WI (*κ* = 0.76) also in assessing extraprostatic extension of PZ cancers [45]. On the other hand, other authors [46] showed that 3D T2WI—volume isotropic T2-weighted acquisition (VISTA)—performed worse than 2D T2WI in assessing extraprostatic extension if including TZ cancers.

It should be pointed that a 3D slab can be generated in a reasonable acquisition time by paying the price of several trade-offs compared to 2D imaging, including lower signal-to-noise ratio, reduced soft tissue contrast, change in image contrast by incorporating T1-weighting as the repetition time is reduced, blurring and loss of resolution even for subtle motion during the scan, and greater motion artefacts in 3D sequences with longer acquisition time [12, 13, 43, 45]. Moreover, although radiologists can perceive 2D and 3D images similarly in terms of image quality, anatomic detail, and tumour conspicuity, the preference for a given T2WI sequence seems based on strong individual preference rather than objective factors [43]. Not surprisingly, 3D T2WI is not yet accepted as a state-of-the-art tool for detecting and staging PCa [13], as exemplified by the recommendation from PI-RADS version 2 to use it as an adjunct to 2D T2WI rather than a stand-alone alternative [10].

### Abbreviated protocols

One might argue that a *less-is-better* strategy in prostate MRI might consist of cutting redundant scans while preserving the informative core of the examination for a certain clinical question. Such an approach showed promising results in screening and staging breast MRI [22] and has been advocated as a mean to improve patients’ compliance, reduce direct costs, and extend availability of the examination.

Kuhl et al. [22] retrospectively compared an abbreviated 3.0 T bpMRI protocol and a full mpMRI in detecting csPCa in 542 men with PSA level > 3 ng/mL and previous negative US-guided systematic biopsy. Abbreviated bpMRI consisted of transverse T2WI and DWI only, with total acquisition time of 8 min 45 s (compared to 34 min 19 s of mpMRI). Using MRI-guided biopsy of category 3–5 findings according to PI-RADS version 2, abbreviated bpMRI detected 138 out of 139 csPCas found with mpMRI, corresponding to a similar cancer detection rate (25.5% versus 25.6%, respectively), and similar sensitivity (93.9% versus 94.6%) and specificity (87.3% versus 84.8%). Inter-reader agreement in attributing category 3 or greater was substantial (*κ* = 0.81), in spite of different readers’ experience.

Abbreviated bpMRI has been advocated as a technical solution to face the increasing demand for prostate imaging [35] or to improve the effectiveness of PSA screening, as recently suggested by some authors using a 5-min MRI protocol [47]. On the other hand, abbreviated prostate MRI needs further validation and should be investigated in terms of cost-effectiveness by balancing saved costs (shorter duration and reading time, lack of contrast injection, early diagnosis of csPCa, and reduced number of biopsies using the MRI-guided approach) versus those that are induced by the procedure (examinations costs and biopsy-related costs) [22]. Furthermore, it is still unknown whether abbreviated MRI is applicable to different clinical scenarios (*e.g.*, cancer staging) and which sequences and planes should be included accordingly.

### Less variability from human readers

**Fig. 5:**
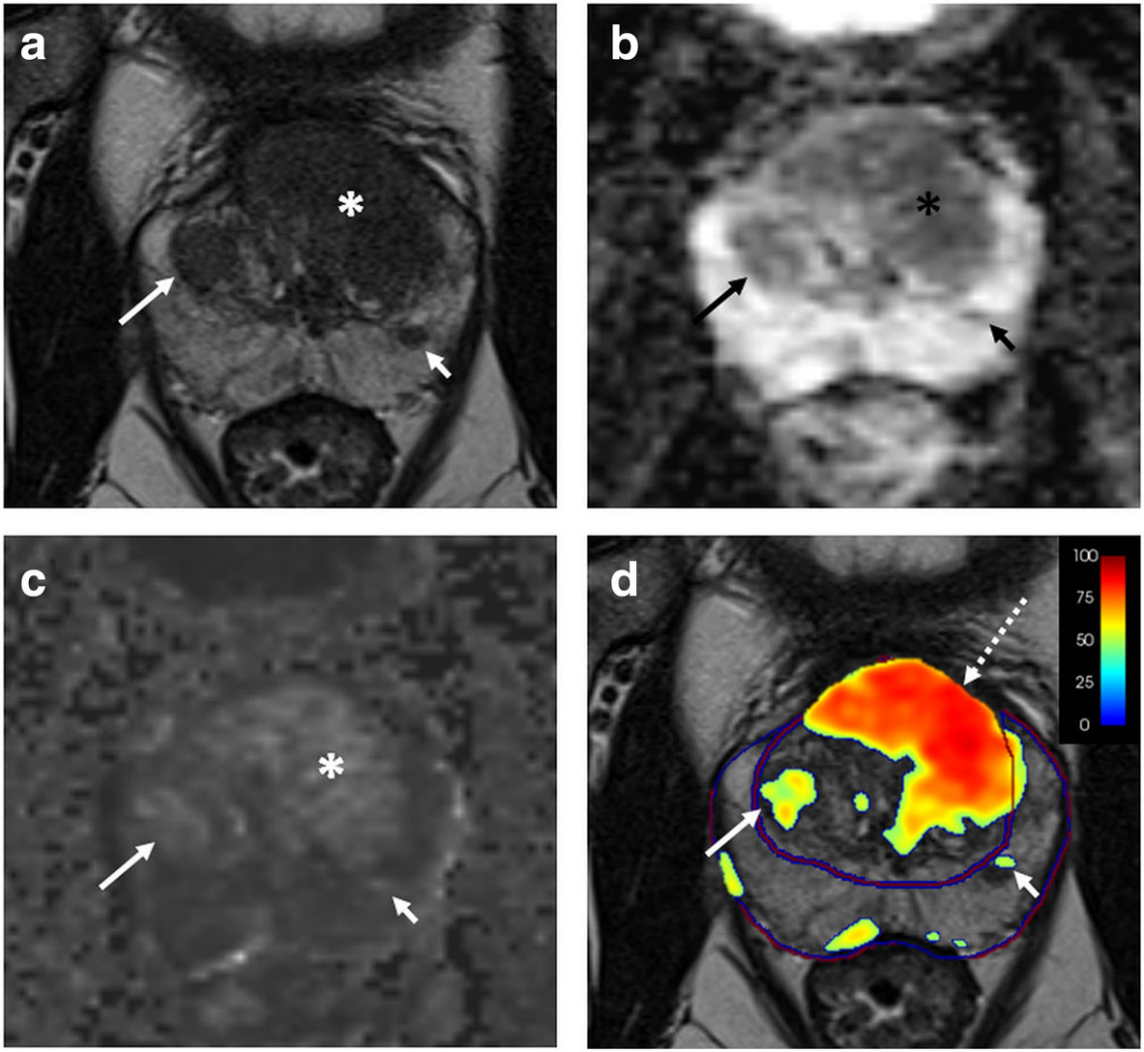
A 66-year-old man with a serum PSA of 14.3 ng/mL. Axial T2-weighted imaging (**a**), ADC map (**b**), and calculated *b* = 1500 s/mm^2^ image (**c**) showed a lesion in the left mid TZ (asterisk in **a**, **b**, **c**). In addition, note a BPH nodule in the right mid TZ (long arrow in **a**, **b**, **c**) and an ectopic BPH nodule in the left mid PZ (short arrow in **a**, **b**, **c**). Computer-aided diagnosis map overlaid on the T2-weighted image (**d**) showed a high cancer probability within the left mid TZ lesion (dashed arrow), whereas it showed low cancer probability within the right mid TZ BPH nodule (long arrow) and the ectopic BPH nodule in the left mid PZ (short arrow). Final pathology revealed a Gleason 7 (3 + 4) prostate cancer within the left mid TZ lesion (image courtesy of Dr. Baris Turkbey and Dr. Stephanie Harmon from Molecular Imaging Program, NCI, NIH, Bethesda, MD, USA)

Standardising the interpretation of prostate MRI with the PI-RADS did not solve the problem of suboptimal inter-reader agreement [19], nor eliminate cancer missing (miss rate up to 30% in some series) [48]. This might partly depend on limitations inherent to the PI-RADS lexicon and interpretation rules [1]. Computer-aided diagnosis (CAD) algorithms have been increasingly studied as a mean to potentially overcome those problems. CAD is a form of machine learning technology, trained on real cases to extract and classify image features, and in turn recognise intermediate- to-high-risk cancers. From a practical point of view, CAD has the task to prompt image markers where csPCa is likely to be present (Fig. 5) [3, 49].

In most studies, this tool has been evaluated as a radiologist’s assistant, with the human reader assuming the final decision on the nature of the findings prompted by CAD. There are plenty of promising results in literature in this regard, as exemplified by a recent multicentre and multireader study in which CAD-assisted readings across different vendors and institutions showed a sensitivity for index-lesions comparable to that of non-assisted readings using PI-RADS version 2 [48]. CAD also helped less experienced readers to diagnose more TZ cancers, and reduced reading time. Whether the increase in sensitivity affects specificity is a matter of debate, with works reporting it as unaltered, improved, or decreased [48, 49]. Controversial results also exist about the accuracy for TZ cancers [48], and the capability to act as a stand-alone reader compared to radiologists of variable experience [49]. Of note, CAD is of particular help to less experienced readers [48] and can increase inter-reader agreement [50].

CAD is a topic of the greatest interest, especially as a tool to improve the cost-effectiveness of prostate cancer screening. The ideal goal of CAD is to assess more csPCa and fewer low-grade cancers [3]. Litjens et al. [51] showed that combining PI-RADS version 1 with CAD improves the differentiation between indolent and aggressive cancers compared to PI-RADS version 1 alone (area under the curve 0.88 versus 0.78, respectively) and that this combination correlates strongly with cancer grade. Moreover, early experience with CAD suggests the potential to better identify, compared to the human eye, the site of csPCa showing higher aggressiveness or true tumour extension. This might translate into guiding the biopsy to more biologically relevant cancer’s foci, as well as a more precise targeting of focal therapy to avoid incomplete ablation [3].

It should be pointed that experiences on CAD differ in terms of algorithms, study populations, standard of reference, use or not use of PI-RADS to interpret images, and definition of csPCa. The role for this tool is far from being firmly established. Importantly, one can ask whether CAD impacts on prostate MRI protocol composition (*e.g.*, bpMRI versus mpMRI). As a matter of fact, CAD includes a variety of technologies, each with its unique approach and reference sequences to analyse (*e.g.*, T2WI alone, DCE alone, T2WI and DWI, T2WI and DCE) [49]. It is difficult to establish whether prostate MRI protocols should be tailored to the available CAD model and concept, or, in contrast, CAD should be modelled on a standard protocol.

## *Different-is-better* strategies

**Fig. 6:**
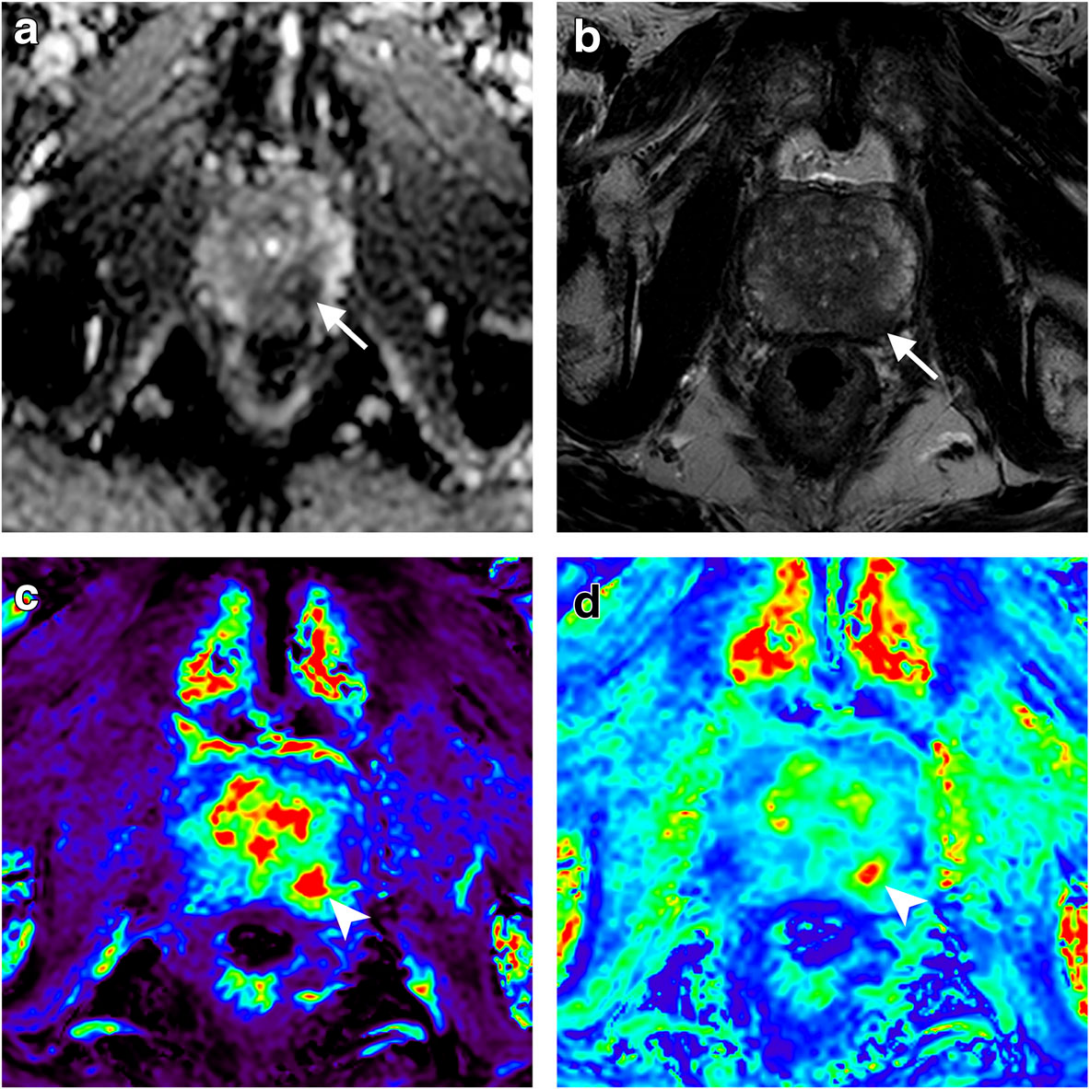
Quantitative DCE imaging in a 71-year-old man undergoing pre-biopsy mpMRI for a left mid PZ cancer (Gleason score 3 + 3 on pathology after radical prostatectomy), showing restricted diffusion on the ADC map (**a**) and hypointensity on T2-weighted imaging (**b**) (arrows). DCE parametric maps obtained using the extended Tofts model showed markedly increased *K*^trans^ (arrowhead in **c**) and *K*_ep_ (arrowhead in **d**) compared to the surrounding PZ (see Table 4 for details)

One might argue that a *less-is-better* strategy can help prostate MRI to gain wider availability for well-established clinical indications. However, there is a parallel pathway of prostate MRI development, searching for objective and reproducible MRI-related biomarkers for the prediction of PCa aggressiveness or overcoming inter-reader variability [52]. Quantitative DWI- or DCE-derived techniques and radiomics are the most exemplificative fields of research in this regard **(**Fig. 6). At the same time, the increasing development of hybrid imaging solutions prompts positron emission tomography/MRI (PET/MRI) as a powerful combination of superior soft tissue contrast with information on tumour biology and/or nodal and bone disease [53]. Once current technical challenges will be solved, one can regard PET/MRI as the ideal all-in-one examination to assess PCa both in locoregional and panoramic terms.

Table 4 shows an overview of goals, derived parameters, and promising achievements of these new strategies as can be appreciated from relevant literature results [52–60]. In general, studies on advanced techniques provide conflicting results and are affected by limitations in design (most of them are based on small single-centre cohorts) and lack of technical standardisation, thus emphasising the need for further and robust validation. Furthermore, advanced techniques imply several challenges in terms of costs, availability of technology and software for analysis, need for expertise, scan duration, and, importantly, the number of additional parameters that might be included in the analysis. While representing an exciting and promising frontier, advanced techniques should be currently regarded as a matter for research with limited probability of being incorporated into clinical practice in the next future.

**Table 4:** Overview of advanced MRI-based techniques for prostate imaging

Type of advanced imaging [reference]	Goals	Derived parameters	Promising achievements
Intravoxel incoherent motion (IVIM) [54]	• To use a biexponential model for separating pure water molecule diffusion from perfusion-related diffusion linked to capillary microcirculation	• Apparent diffusion coefficient (ADC) • Pure molecular diffusion (D) • Perfusion-related parameters such as D* and f	• Increased accuracy in detecting PCa compared to the mono-exponential model, though with no added value in the TZ • Differentiation of high-grade versus low-grade tumours, especially when using D
Diffusional kurtosis imaging (DKI) [52, 54]	• To account for non-Gaussian distribution of water molecules motion due to heterogeneous microenvironments with many or large interfaces (*e.g.*, intracellular structures and organelles) • To better exploit tissue microstructural complexity • To better represent water diffusion within the intracellular compartment, and in turn better represent tissue cellularity	• Diffusion coefficient *D*_app_ (corrected for observed non-Gaussianity) • Apparent diffusional kurtosis *K*_app_ (a dimensionless measure of the deviation of tissue diffusion from a Gaussian pattern)	• Better than DWI in assessing PCa and in differentiating low- versus high-grade tumours
Diffusion tensor imaging (DTI) [56]	• To account for the degree of anisotropy affecting water diffusion	• ADC • Fractional anisotropy • DTI tractography	• Correlation with tumour aggressiveness and tissue composition
Restriction spectrum imaging (RSI) [57]	• To collect diffusion data with multiple gradient directions and *b* values, in association with a linear mixture model to resolve a spectrum of length scales, and acquisition of geometric information • To separate intracellular from extracellular signal, and in turn better reflect tissue cellularity • To account for underlying geometry information	• RSI cellularity index	• Added value compared to mpMRI in detecting PCa • Close correlation with Gleason score • Correcting for geometric distortion in targeted biopsy of small volume lesions • RSI has the potential to be normalised in a machine- and technique-independent way
Quantitative dynamic contrast-enhanced (DCE) imaging [58, 59]	• Deriving quantitative parameters to describe tissue vascularisation and blood flow in the normal prostate or PCa	They depend on the pharmacokinetic model used. Examples: • Transfer constant (*K*^trans^): exchange constant between blood plasma and extravascular extracellular space • Rate constant (*K*_ep_): exchange constant between extravascular extracellular space and blood plasma	• Improving cancer detection, localisation, and staging • Assessment of biological aggressiveness and prognosis • Increased sensitivity for recurrent cancer after radiation therapy, radical prostatectomy, or high-intensity-focused ultrasound • Monitoring the effects of hormone therapy or antiangiogenic drugs
Radiomics [60]	• To extract quantitative information from medical images (statistics, metrics, descriptors), thus accounting for biological heterogeneity of disease	A large variety of features describing: • Intensity • Texture • Shape	• Automatic or semiautomatic segmentation of the prostate for radiation therapy planning, biopsy preparation, volume estimation, and PCa localisation • Detection and risk stratification in active surveillance • Pathological grade prediction • Identification of biologically relevant targets for biopsy • Radiogenomics
PET/MRI [53]	• To combine superior soft tissue contrast of MRI with panoramic biologic information from PET	A variety of radiotracers are used in PET/MRI, including: • [^18^F]-NaF for bone metastatic disease • [^11^C]-choline for recurrent disease • [68Ga]-PSMA-HBED-CC (for staging, recurrence, and treatment response assessment)	• Improved diagnosis compared to MRI alone • Improved accuracy in detecting and characterising bone disease compared to PET/CT • Improved detection of local recurrence compared to PET/CT alone

*ADC* apparent diffusion coefficient, *CT* computed tomography, *D* diffusion, *DTI* diffusion tensor imaging, *DWI* diffusion-weighted imaging, *mpMRI* multiparametric MRI, *MRI* magnetic resonance imaging, *PCa* prostate cancer, *PET* positron emission tomography, *PSMA* prostate-specific membrane antigen, *RSI* restriction spectrum imaging, *TZ* transition zone

## Conclusions

The evolution of PI-RADS testifies that prostate MRI technique and interpretation were simplified over the last years, in line with the need to support the ever-increasing expansion of the examination in clinical practice, and achieve robust standardisation across different centres and readers. Although well validated in terms of diagnostic accuracy, state-of-the-art prostate MRI is based on a multiparametric approach combining anatomic and functional imaging, which represents a costly, time consuming, and somewhat poorly patient-centred standard.

Several *less-is-better* strategies have been proposed to overcome the limitations of mpMRI. Of them, bpMRI is becoming increasingly popular for detection/localisation and staging of PCa. At the same time, parameters derived from advanced techniques are a matter for intensive research, especially as potentially reproducible imaging biomarkers to be included, in the future, within a revised multiparametric standard. Both approaches imply the need for refined interpretation rules compared to those developed by the PI-RADS using mpMRI as a reference, thus emphasising the strict correlation between image acquisition, interpretation, and reporting.

A crucial point for the evolution of prostate MRI is how to accomplish for patient needs and the increasing demand for the examination. The scenario in which prostate MRI is performed will probably make the difference. Indeed, while *less-is-better* strategies are promising for cancer detection, localisation, and staging in clinical practice, *different-is-better* strategies better reflect the context of academic centres, in which the investigation of multiple parameters is supported by research activity.

## Abbreviations

2D: Two-dimensional
3D: Three-dimensional
bpMRI: Biparametric MRI
CAD: Computer-aided diagnosis
csPCa: Clinically significant PCa
DCE: Dynamic contrast enhanced
DWI: Diffusion-weighted imaging
ESUR: European Society of Urogenital Radiology
mpMRI: Multiparametric MRI
MRI: Magnetic resonance imaging
MRSI: Magnetic resonance spectroscopic imaging
PCa: Prostate cancer
PET: Positron emission tomography
PI-RADS: Prostate Imaging Reporting and Data System
PSA: Prostate-specific antigen
PZ: Peripheral zone
T2WI: T2-weighted imaging
TZ: Transition zone
